# Polyacrylonitrile Nanofiber-Based Quartz Crystal Microbalance for Sensitive Detection of Safrole

**DOI:** 10.3390/s18041150

**Published:** 2018-04-10

**Authors:** Aditya Rianjanu, Roto Roto, Trisna Julian, Shidiq Nur Hidayat, Ahmad Kusumaatmaja, Eko Agus Suyono, Kuwat Triyana

**Affiliations:** 1 Department of Physics, Universitas Gadjah Mada, Sekip Utara, Yogyakarta 55281, Indonesia; trisna.julian@mail.ugm.ac.id (T.J.); shidiq.nurhidayat@mail.ugm.ac.id (S.N.H.); ahmad_k@ugm.ac.id (A.K.); 2 Department of Chemistry, Universitas Gadjah Mada, Sekip Utara, Yogyakarta 55281, Indonesia; roto05@ugm.ac.id; 3 Nanomaterial Research Group, Universitas Gadjah Mada, Sekip Utara, Yogyakarta 55281, Indonesia; 4 Department of Biology, Universitas Gadjah Mada, Sekip Utara, Yogyakarta 55281, Indonesia; eko_suyono@ugm.ac.id

**Keywords:** polyacrylonitrile nanofiber, gas sensor, ecstasy, MDMA, safrole, quartz crystal microbalance

## Abstract

Safrole is the main precursor for producing the amphetamine-type stimulant (ATS) drug, *N*-methyl-3,4-methylenedioxyamphetamine (MDMA), also known as ecstasy. We devise a polyacrylonitrile (PAN) nanofiber-based quartz crystal microbalance (QCM) for detecting safrole. The PAN nanofibers were fabricated by direct electrospinning to modify the QCM chips. The PAN nanofiber on the QCM chips has a diameter of 240 ± 10 nm. The sensing of safrole by QCM modified with PAN nanofiber shows good reversibility and an apparent sensitivity of 4.6 Hz·L/mg. The proposed method is simple, inexpensive, and convenient for detecting safrole, and can be an alternative to conventional instrumental analytical methods for general volatile compounds.

## 1. Introduction

According to the recent World Drug Report, the global abuse of amphetamine-type stimulus (ATS) drugs continues to increase. In particular, the abuse of *N*-methyl-3,4-methylenedioxyamphetamine (MDMA), well known as ecstasy, has been growing rapidly [1], despite its serious unhealthy side effects [2]. The drug is produced in Europe, Asia, North America, Oceania, and South America. Since 2010, the quantity of drugs seized globally has averaged around 4–5 tons. In Indonesia alone, the annual confiscated crystal meth has increased significantly from 0.1 to 1.3 tons in the last eight years [3]. Detecting and curbing the illegal trade remain a problem faced by law enforcement.

Precursors commonly used to produce amphetamine-like drugs include piperonal, safrole, and iso-safrole [3]. They are also used in the chemical and pharmaceutical industries, making them prone to black market trading. Safrole is a pale-yellow liquid precursor that has been smuggled throughout South Asia.

There are many conventional analytical methods for detecting drugs, which are mainly chromatographic-based, such as gas chromatography (GC), liquid chromatography (LC), and liquid chromatography coupled with mass spectrometry (LC-MS) [4,5]. Although these methods are very sensitive and selective, they are costly, not handy, and difficult to operate. Therefore, there is a need for a portable, easy-to-use, and cheap analytical instrument for drug detection.

In recent years, the development of gas sensors to detect volatile organic compounds (VOCs) in the atmosphere is rapidly growing. Many gas sensors have been developed based on sensing chip-analyte interactions such as photoelectric [6,7], resistive [8,9], optical [10], amperometric [11], and acoustic wave interactions [12]. Quartz crystal microbalance (QCM) is one example of acoustic wave sensor platform based on acoustic-electric effect and mass deposition. The QCM sensor offers accurate, real-time, and convenient detection of gases or vapors [12,13] and humidity [14]. Nanostructured materials have become one of the most studied materials for devices, especially those used for gas sensing [15,16,17]. These materials, including nanofibers, have a large specific surface area, high porosity, and interconnected porous structures [18,19,20], which are advantageous for preparing gas sensing devices. The gas sensor prepared by nanostructured materials has a better sensitivity [21,22,23] because of the increase in the specific surface area of the materials [22,23].

Several studies have been conducted to develop mobile, easy-to-use, and cheap analytical instruments for detecting safrole. Pinalli et al. developed an amphetamine precursor detection method using quinoxaline-bridge cavitands [24], whereby the detection was confirmed by GC-MS analyses. Hackner et al. reported an analytical instrument to detect amine-containing drugs with surface ionization detection method [25]. They attempt to demonstrate that illicit amine-containing drugs can be sensitively detected using flash evaporation directly into the air gap of a surface ionization detector, interfaced to gas chromatographic (GC).

In this study, we report on safrole sensing based on the QCM method using polyacrylonitrile (PAN) nanofibers produced directly on the sensing surface. This nanofiber is mechanically stable, water-insoluble, and hydrophobic. It is chemically unique since it has lone pair electrons, and its hydrophobicity is essential since QCM gas sensor is heavily interfered by water vapor. The lone pair electrons of PAN can better interact with the electron-deficient functional groups in the analytes by intermolecular bond.

## 2. Materials and Methods

### 2.1. Materials

Polyacrylonitrile with molecular weight of 150,000 g/mol was supplied by Sigma-Aldrich (St. Louis, MO, USA). Non-ionic surfactant Triton X-100 and *N*,*N*-dimethyl formamide (DMF) were purchased from Merck (Darmstadt, Germany). Safrole with 96% purity, which was used as the main analyte, was supplied by the Indonesian National Police Jakarta. Other chemicals including ammonia, formaldehyde, acetone, and toluene were purchased from Merck, Germany. The AT-Cut QCM sensors with the gold electrode and 10 MHz base resonant frequency were purchased from OpenQCM, Novaetech. R&D, Napoli, Italy. All chemicals were used as received without further purification.

### 2.2. Nanofiber Preparations

**Figure 1:**
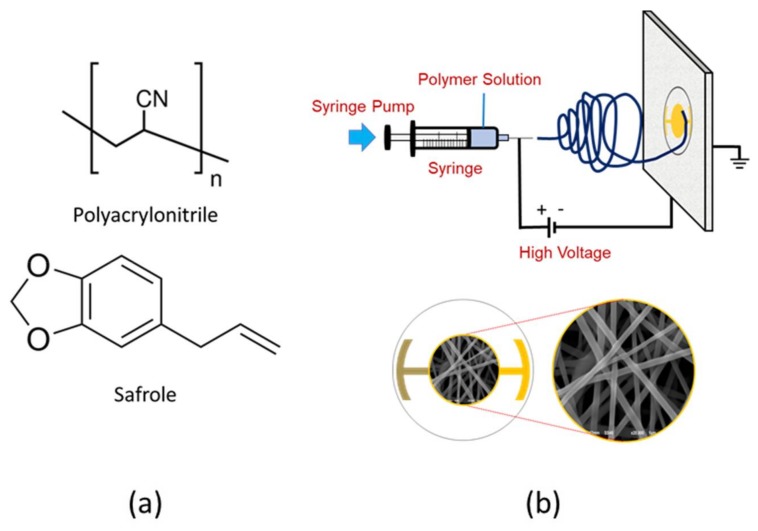
(**a**) Chemical structure of polyacrylonitrile (PAN) and safrole; (**b**) schematic illustration of electrospinning set-up and nanofiber image deposited on the quartz crystal microbalance (QCM) surface.

Polyacrylonitrile solution was prepared by dissolving 0.60 g PAN in the 10 mL DMF, followed by stirring at 1000 rpm for 24 h. A small amount (1%) of non-ionic surfactant, Triton X-100, was added to the PAN solution to lower its surface tension. Mechanical stirring at 900 rpm was extended for 30 min to obtain a homogeneous solution in the presence of a surfactant. The schematic of the electrospinning set-up and chemical structure of PAN and safrole are shown in Figure 1.

The PAN solution was transferred into 10 mL plastic syringes for electrospinning, which was performed at a DC voltage of 10 kV with a predetermined 15 cm distance between the tip and collector. The QCM gold plate was used as fiber collector where the outer area was coated with aluminum foil. The electrospinning time was varied from 10 to 40 s. The nanofiber film was dried in a desiccator for 12 h to evaporate any residual solvent before further treatment.

### 2.3. Sensor Apparatus for Safrole Detection

**Figure 2:**
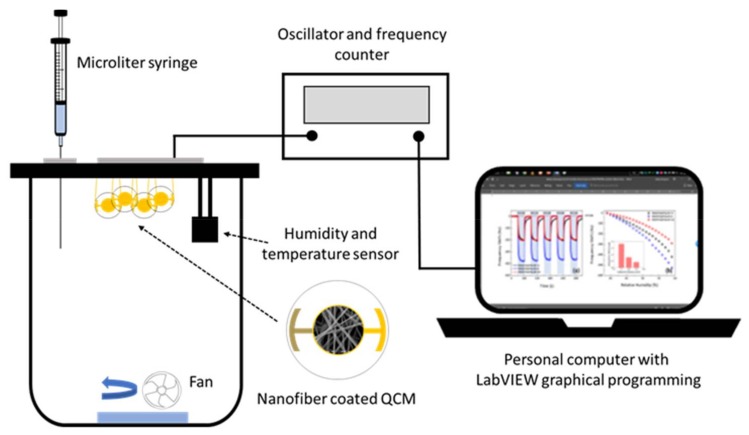
Schematic illustration of safrole QCM gas sensing configuration.

A schematic diagram of a static-type safrole vapor testing system is shown in Figure 2. The PAN nanofiber-based QCM sensor was installed in the testing chamber (1.25 L), which was kept at ambient temperature. The temperature and the relative humidity of the sensing chamber were controlled at (33 ± 1) °C and 55–65% RH, respectively. Sensirion SHT-31 was used as temperature and relative humidity sensors. A microliter syringe (1–10 µL, Hamilton Model 701 RN SYR) was used to inject the analyte. The frequency counter of the QCM sensor was used to measure the resonance frequency, whereby the frequency changes based on the mass shift of the sensor. The measured data were recorded by a personal computer with graphical programing language LabVIEW. The ambient air was used to desorb the analyte from the sensing membranes. The concentration of injected safrole in the vapor chamber was calculated in mg/L using its density, percent purity, and volume.

### 2.4. Sample Characterization

The nanofiber microstructure was analyzed by JEOL JSM–6510 scanning electron microscope (SEM). The samples were mounted on metal stubs and sputter-coated with platinum for 120 s with JEOL JEC−3000FC auto fine coater.

## 3. Results and Discussion

### 3.1. Nanofiber Morphology

**Figure 3:**
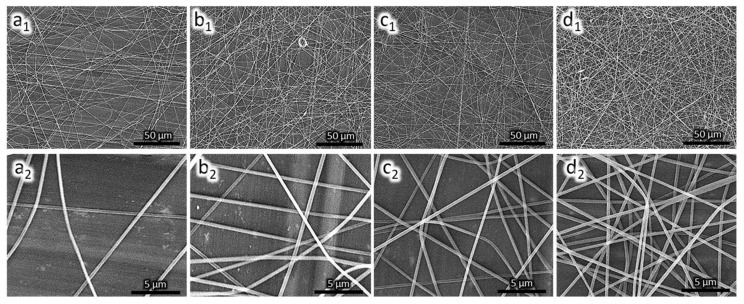
SEM image of PAN nanofiber on QCM surface; (**a**) PAN-NF 1; (**b**) PAN-NF 2; (**c**) PAN-NF 3; and (**d**) PAN-NF 4.

Figure 3 shows the PAN nanofiber microscopic image at different magnifications. The nanofibers are smooth and continuous with an average diameter of (242 ± 10) nm. The deposition time affects the quantity of nanofiber deposited on the QCM substrate. The deposition rate remained constant. For a long deposition time, more nanofibers are deposited on the surface as indicated by an increase in frequency drop. The mass loading on the QCM substrate follows Sauerbrey equation [26] as shown in Equation (1).

(1) $$\Delta f=-\frac{2f_{0}^{2}}{A\sqrt{\rho_{q}\mu_{q}}}\;\Delta m$$

where Δf represents the frequency shift of crystal QCM (Hz), $f_{0}$ is the base resonance frequency (10 MHz), Δm is the mass change (g), A is the electrode surface area (0.283 cm^2^), and $\mu_{q}$ and $\rho_{q}$ are the shear modulus and density of quartz crystal (2.947 × 10^11^ g cm^−1^ s^−2^) and (2.648 g cm^−3^), respectively.

The mass of PAN nanofibers deposited on the QCM chip is shown in Table 1. The mass loadings of PAN nanofiber were calculated using Equation (1) as 0.174, 0.578, 1.041 and 1.734 µg, for deposition times of 10, 20, 30 and 40 s, respectively. For convenience, we assigned the trials as indicated. The increase of mass loading is linearly dependent on the increase of deposition time. The mass loading on the active layer, for rigid film, affects gas sensor response [27].

**Figure 4:**
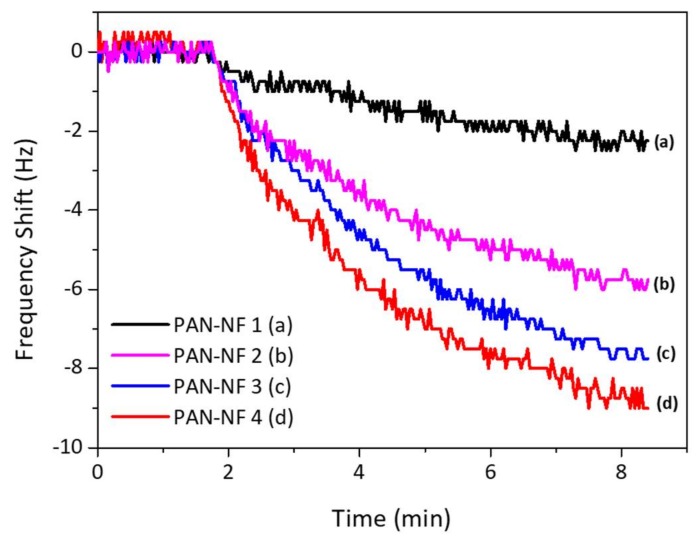
The QCM response to 1 mg/L safrole vapor.

The frequency response of the fabricated sensor is shown in Figure 4. The response time is about 8–10 min. As the mass deposition increases, the frequency shift gets higher, allowing an assumption that the surface area of the PAN nanofiber sensor increases with increase in the PAN nanofiber loading mass as stated in literature [23]; hence, the surface area becomes the vital parameter in developing a gas sensor based on QCM technique. However, the mass loading affects the maximum electrode contact area fully covered by the nanofiber, as it limits the response of the sensor.

### 3.2. PAN Nanofiber Gas Sensor Response and Sensitivity

Figure 4 shows the response of QCM to 1 mg/L of safrole vapor, where the PAN nanofibers are prepared at different times of electrospinning. The measurement was carried out at ambient temperature and humidity of ~60% RH. The frequency shift increases with PAN nanofiber loading, and the surface area of the nanofibers increases with mass loading of the PAN, which would improve the contact area between the active layer and analytes. The porous nature of the nanofibers interacts with safrole molecule.

The data also show that the response time is quite slow, as response starts to steady after 7 min. This relatively slow response is probably due to the low vapor pressure of the safrole (0.0706 mm Hg at 25 °C), which makes it difficult to evaporate at room temperature but good enough to be detected with the sensing system.

**Figure 5:**
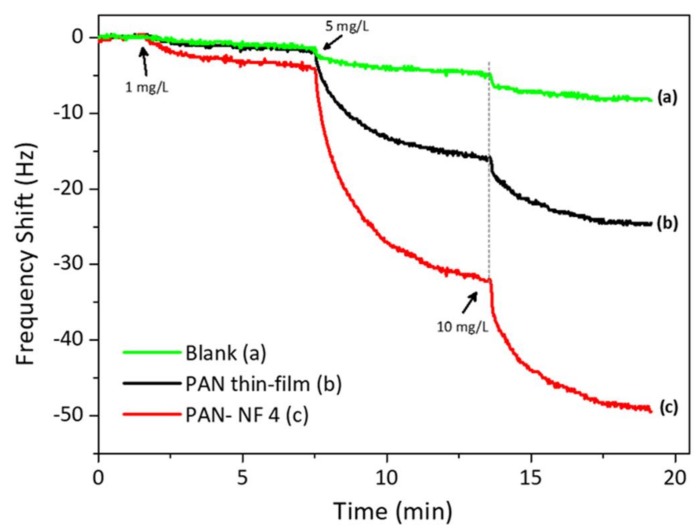
The QCM response to safrole of bare, modified with PAN by spin-coating and modified with PAN by electrospinning.

To ascertain the effect of surface area on the response size, we also coated the QCM substrate with PAN by spin-coating for 30 s at 1000 rpm. The data are depicted in Figure 5. The QCM coated by spin-coating resulted in a mass of 2.2 µg, which is higher than that coated by electrospinning. For reference, uncoated QCM gave a small response to the analyte. On the other hand, the QCM coated with PAN nanofibers via electrospinning interacted strongly with safrole vapor, with a frequency shift of 5 Hz for each 1 mg/L safrole vapor. It produced a mass shift two-fold larger than that produced by the spin-coated QCM. Thus, it is evident that QCM modified with PAN nanofibers via electrospinning has better sensitivity than that modified by spin-coating.

**Figure 6:**
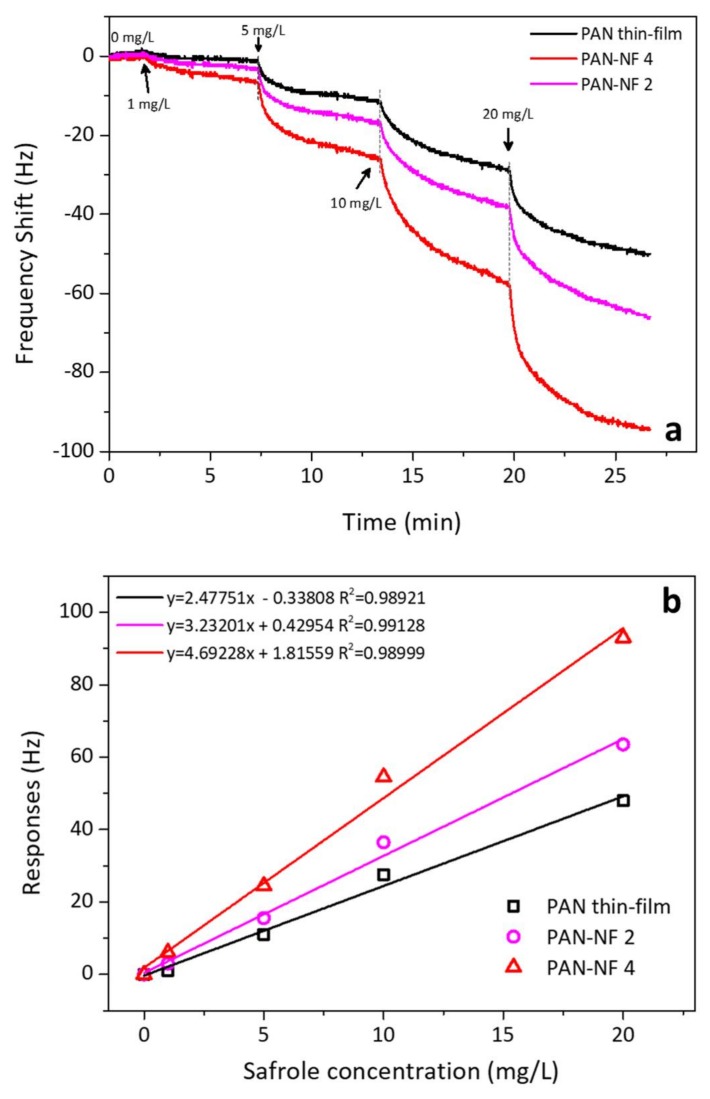
(**a**) QCM response to the various analyte concentrations; (**b**) QCM response after 5 min of contact with different analyte concentrations. The slope of the curve indicates the selectivity of the method.

Figure 6a shows the frequency responses for QCM when modified with spin-coating and electrospinning. The concentration of the safrole vapor was predetermined from 1 to 20 mg/L. Upon safrole injection into the sensing chamber, the response was sharp and steady after several minutes. The QCM sensor coated with PAN-NF 4 can detect safrole of 1, 5, 10 and 20 mg/L, corresponding to a frequency shift of 8, 26, 55 and 95 Hz, respectively. At low analyte concentration, we observed that the response of the PAN nanofiber sensor gradually increased with the safrole vapor concentration. However, the high concentration of safrole vapor resulted in less response, which is due to saturation of the polymer surface covered by the analyte.

Figure 6b shows the sensitivity of the QCM to the analyte. The slope increases with the film thickness. The sensitivity shifts from 2.5 to 3.2 and 4.7 Hz·L/mg for the spin-coating films PAN-NF 2 and PAN-NF 4, respectively, while the literature sensitivity was found about 5 L/mg [28]. The correlation constant (*χ*^2^) of the analyte concentration and the response is 0.98921 or higher, which is good. The sensitivity of the QCM modified with PAN nanofiber improves with increase in electrospinning time. The increase in the response is believed to be due to improved surface area of the nanofiber, which can be attributed to the high porosity of PAN nanofibers. The specific surface area of nanofiber membranes can improve when the diameters of polymer nanofibers are small [23]. The simulated sample was also analyzed by gas chromatography–mass spectrometry, which had safrole content of 96% to give estimated bias of 4–5%.

### 3.3. Reversibility of PAN Nanofiber QCM Sensor

**Figure 7:**
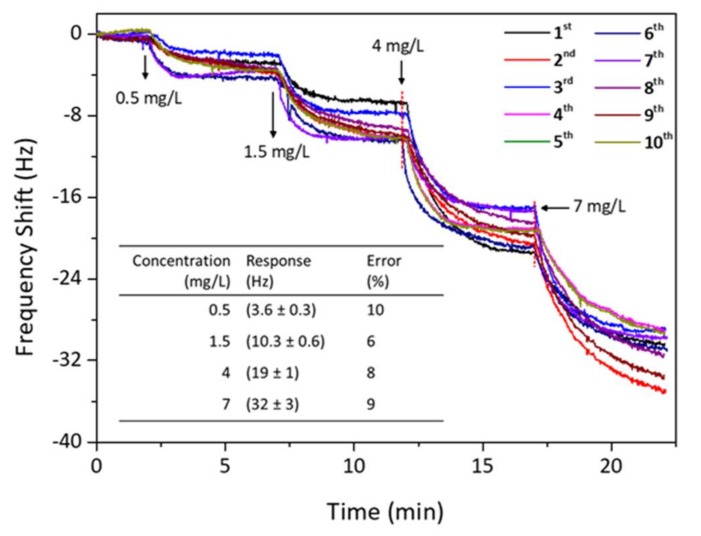
Reversibility testing for PAN-NF 4 nanofiber QCM sensors upon exposure to increasing the safrole concentrations. (Inset: systematic error of PAN-NF 4 sensor from 10-time measurement; the response is taken at *t* = 5 min after each injection).

Figure 7 shows the reversibility trial for the QCM sensor of PAN-NF 4 to detect safrole vapor. When the response of the sensor chip reached equilibrium, fresh and dry ambient air was introduced to the detection chamber to flush absorbed safrole until desorption was complete. The sensor was again exposed to the safrole vapor with the same concentration as before. It shows that adsorption–desorption cycles are reproducible after re-exposure to the analyte. The frequency change returns to its original value after purging the sensor chip with dry and fresh air. The sensing response was nearly repeatable and reproducible after ten consecutive sample injections, indicating that the proposed QCM method is reversible in detecting safrole vapor.

### 3.4. Selectivity of Safrole Sensing Over Other Gas

**Figure 8:**
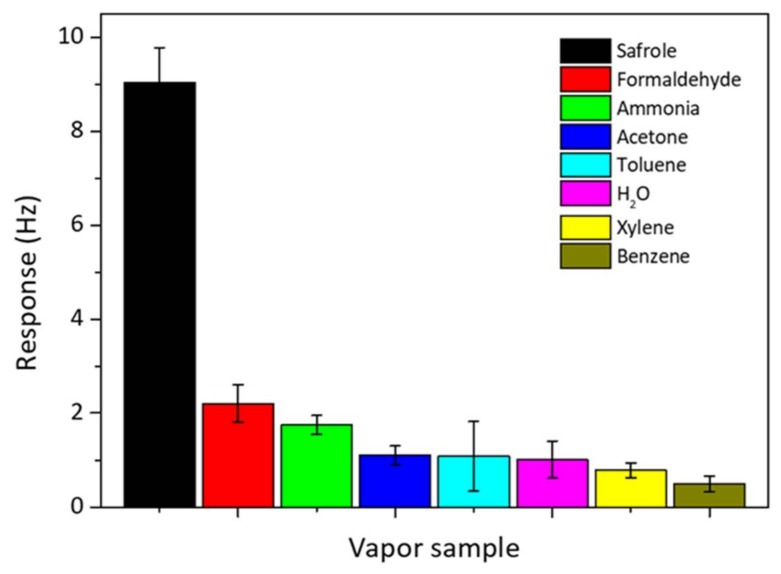
Selectivity of PAN nanofiber-coated QCM sensor over other analytes. The analyte concentration is predetermined at 1 mg/L.

The selectivity of the QCM sensor is important for real and practical detection of safrole vapor. The selectivity test was done by exposing the QCM chip modified with PAN nanofibers to various VOCs of 1 mg/L commonly present in the atmosphere, and they include ammonia (25% in aqueous solution), formaldehyde (37% in aqueous solution), acetone, H_2_O, benzene, toluene, and xylene. The results are shown in Figure 8. The responses for these organic vapors are smaller than that for safrole; since the PAN nanofibers selectively interact with safrole. At concentration 1 mg/L, safrole gives the highest response of frequency shift up to 9 Hz, whereas other VOCs give a response of 2.2 Hz or lower.

The high interaction between the nanofibers and safrole suggests that the sensing surface is more selective toward safrole than the other tested vapors. Previous works show that the high affinity of the QCM active layer toward specific analytes could result in a great response or high selectivity [29,30]. The QCM sensor modified with PAN by electrospinning has a better selectivity to safrole than to the other tested analytes. The developed QCM system for safrole detection could be of significant application in curbing illegal drug production and abuse.

### 3.5. Sensing Mechanism

**Figure 9:**
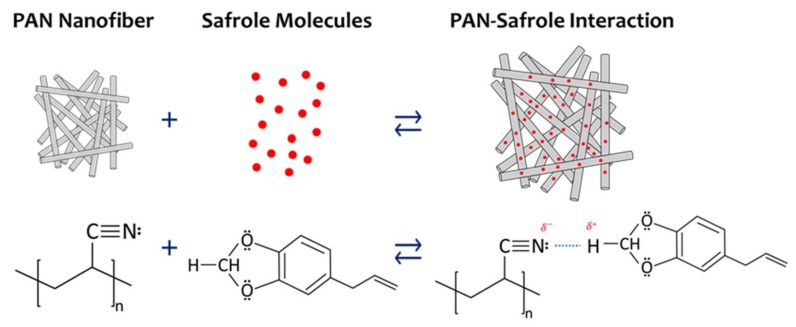
Schematic representation of the interaction mechanism between safrole and PAN nanofiber.

Figure 9 illustrates a probable safrole sensing mechanism by the PAN nanofiber QCM-based sensor. Each subunit of PAN has a nitrogen atom that acts as Lewis base. One lone electron pair in each PAN subunit is believed to interact strongly with two protons located between two oxygen atoms of safrole. These two hydrogen atoms are the most electron-deficient ones in the structure that acts as strong Lewis acid. The intermolecular hydrogen bonding between the hydrogen atoms and the nitrogen atom is strong, which leads to the significant frequency shift. Safrole may also have a weak interaction with PAN through other intermolecular interactions such as hydrophobic–hydrophilic, dipole–dipole, and π–π interactions. Moreover, the intermolecular interactions of the other tested VOCs are not as strong as that of safrole.

Many studies have reported that the unique structure of nanofibrous material with high porosity could be advantageous for achieving strong and rapid QCM responses to analytes [31,32]. A large shift in frequency indicates a strong interaction between safrole and PAN nanofibers. The large surface area, high porosity, and hierarchical structure of PAN nanofiber provide high diffusion for the analyte, which results in a big frequency shift. Furthermore, the high surface area of the PAN nanofibers allows safrole molecules to diffuse deep into the film, which helps increase the frequency response. The 3D porous structure alone is not enough to produce both excellent sensitivity and selectivity. In our next study, we will focus on in-depth investigation of the sensing mechanism of a polymer-modified QCM sensor.

## 4. Conclusions

A new PAN nanofiber-based QCM sensor was successfully fabricated by electrospinning. The PAN nanofibrous structure clearly improved the sensor performance compared to the thin-film PAN because of a larger surface area and higher porosity. The 3D structure of the PAN nanofibrous membranes provided a permeable space supported by a possible dipole–dipole interaction between PAN and safrole, which together imparted the sensor with good sensitivity and stable properties. The proposed system has good reversibility, high selectivity, and excellent sensitivity (4.6 Hz·L/mg with *χ*^2^ = 0.99) for detecting safrole. Hence, it could be used for routine detection of safrole. The sensing mechanism, however, may be the subject of further investigation. This work may promote a new strategy for sensing development of drugs, especially MDMA and other ATS derivatives.

## Figures and Tables

**Table 1:** Electrospinning parameters and frequency shift after coating with PAN nanofiber.

Trial	Time (s)	Frequency Shift (Hz)	Deposited Mass (µg)
PAN-NF 1	10	150	0.2
PAN-NF 2	20	500	0.6
PAN-NF 3	30	900	1.0
PAN-NF 4	40	1500	1.7
